# Supplementary material for: Transcript Markers from Urinary Extracellular Vesicles for Predicting Risk Reclassification of Prostate Cancer Patients on Active Surveillance
Source: Cancers (Basel). 2024 Jul 4;16(13):2453. doi: 10.3390/cancers16132453 (PMC11240337; doi:10.3390/cancers16132453)
Supplement: Supplementary file 1 [file cancers-16-02453-s001.zip › cancers-3061527-supplementary.pdf]

Transcript Markers from Urinary Extracellular Vesicles for Predicting Risk Reclassification of Prostate Cancer Patients on Active Surveillance

Kati Erdmann, Florian Distler, Sebastian Gräfe, Jeremy Kwe, Holger H. H. Erb, Susanne Fuessel, Sascha Pahernik, Christian Thomas and Angelika Borkowetz

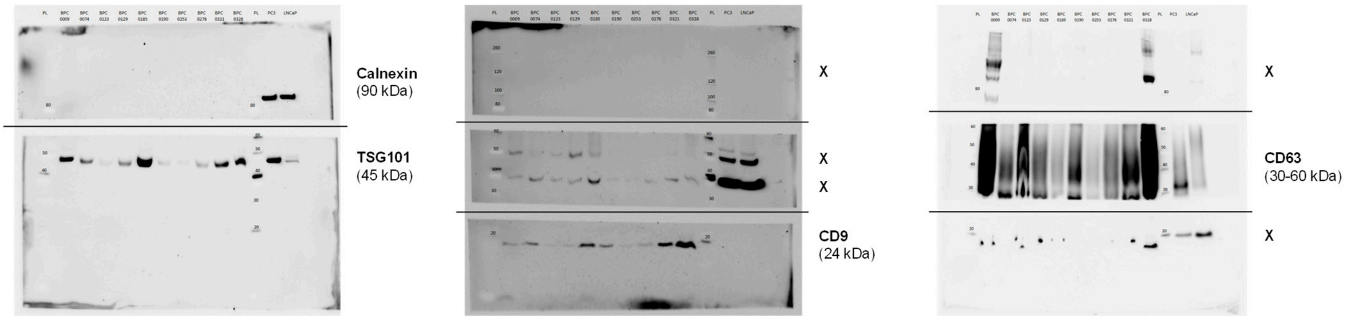

Figure S1. The uncropped Western Blots of Figure 1. The membranes were cut horizontally in order to detect several proteins simultaneously (indicated by black line). Please note, that additional proteins were evaluated, which are not depicted in the manuscript (marked by X). PL: protein ladder in kDa.

Table S1. Transcripts including their NCBI Gene ID quantified by qPCR using TaqMan Gene Expression Assays (Thermo Fisher Scientific, Darmstadt, Germany), which contained the appropriate primers and probes.

Transcript	NCBI Gene ID	Name	Assay ID
AMACR	23600	alpha-methylacyl-CoA racemase	Hs01091292_m1
BCL2	596	BCL2 apoptosis regulator	Hs01048932_g1
BCL-XL = BCL2L1	598	BCL2 like 1	Hs00236329_m1
CCND1	595	cyclin D1	Hs00765553_m1
DLX1	1745	distal-less homeobox 1	Hs00698288_m1
ERG	2078	ETS transcription factor ERG	Hs01554629_m1
EZH2	2146	enhancer of zeste 2 polycomb repressive complex 2 subunit	Hs00544830_m1
HPN	3249	hepsin	Hs01056332_m1
HOXC6	3223	homeobox C6	Hs00171690_m1
KLK2	3817	kallikrein related peptidase 2	Hs00428384_g1
KLK3 = PSA	354	kallikrein related peptidase 3	Hs02576345_m1
MALAT1	378938	metastasis associated lung adenocarcinoma transcript 1	Hs00273907_s1
MCL1	4170	MCL1 apoptosis regulator, BCL2 family member	Hs01050896_m1
NEAT1	283131	nuclear paraspeckle assembly transcript 1	Hs03453535_s1
PCA3	50652	prostate cancer associated 3	Hs01371939_g1
PCAT1	100750225	prostate cancer associated transcript 1	Hs04275836_s1
PCAT14	101978785	prostate cancer associated transcript 14	Hs04941925_m1
PCAT29	104472713	prostate cancer associated transcript 29	Hs04942272_m1
PCGEM1	64002	PCGEM1 prostate-specific transcript	Hs01369007_m1
PPIA [a]	5478	peptidylprolyl isomerase A	Hs99999904_m1
Prostein = SLC45A3	85414	solute carrier family 45 member 3	Hs01026319_g1
PSGR = OR51E2	81285	olfactory receptor family 51 subfamily E member 2	Hs04231197_m1
PSMA = FOLH1	2346	folate hydrolase 1	Hs00379515_m1
RPLP0 [a]	6175	ribosomal protein lateral stalk subunit P0	Hs00420895_gH
SChLAP1	101669767	SWI/SNF complex antagonist associated with prostate cancer 1	Hs04968419_m1
SPDEF	25803	SAM pointed domain containing ETS transcription factor	Hs00171942_m1
STAT3	6774	signal transducer and activator of transcription 3	Hs00374280_m1
STAT5A	6776	signal transducer and activator of transcription 5A	Hs00559637_g1
STAT5B	6777	signal transducer and activator of transcription 5B	Hs00560026_m1
TBP [a]	6908	TATA-box binding protein	Hs00427620_m1
TMPRSS2	7113	transmembrane serine protease 2	Hs01122322_m1
TRPM8	79054	transient receptor potential cation channel sub-family M member 8	Hs01066596_m1

[a] Transcripts used as references for qPCR normalization. NCBI: National Center for Biotechnology Information.

Table S2. Relative expression levels of transcripts in uEV from stable and reclassified patients at control biopsy and respective fold changes.

Transcript	Median relative expression levels (x 10^{-3})		Fold Change (Reclassified vs Stable)	p Value
	**Stable n = 41**	**Reclassified n = 31**		
AMACR	148.60	201.10	+1.35	**0.024**
BCL2	7.39	7.74	+1.05	0.384
BCL2L1 = BCL-XL	589.90	579.30	-1,02	0.396
CCND1	291.20	322.60	+1.11	0.557
DLX1 [a]	0.00 (mean: 0.40)	0.18 (mean: 0.56)	NE (+1.40)	0.197
ERG	0.58	0.85	+1.47	0.535
EZH2	5.58	5.92	+1.06	0.422
HPN	51.60	60.53	+1.17	**0.069**
HOXC6	3.30	4.81	+1.45	0.104
KLK2	709.60	597.80	-1,19	0.125
KLK3 = PSA	947.70	850.00	-1,11	0.557
MALAT1	35.59	23.39	-1,52	**0.099**
MCL1	395.70	388.60	-1,02	0.804
NEAT1	59.04	37.12	-1,59	0.117
PCA3	403.60	421.40	+1.04	**0.093**
PCAT1	10.52	6.62	-1,59	0.428
PCAT14	27.31	32.40	+1.19	0.457
PCAT29	1.72	2.90	+1.68	**0.067**
PCGEM1	130.30	129.10	-1,01	0.901
Prostein = SLC45A3	409.50	371.00	-1,10	0.173
PSGR = OR51E2	81.09	91.86	+1.13	0.442
PSMA = FOLH1	103.10	99.25	-1,04	0.955
SChLAP1	6.02	7.52	+1.25	0.409
SPDEF	1396.00	1497.00	+1.07	0.527
STAT3	190.00	188.10	-1,01	0.422
STAT5A	3.22	2.92	-1,10	0.964
STAT5B	42.86	40.37	-1,06	0.435
TMPRSS2	678.80	642.40	-1,06	0.287
TRPM8	371.60	333.20	-1,12	0.910

Depicted are the median relative transcript levels (normalized to geoM of PPIA, RPLP0 and TBP) in patients with stable disease and risk reclassification as well as the fold change in the reclassified group compared to the stable group ([a] For DLX, mean values were also used to calculate the fold change). *P* values were calculated by the Mann-Whitney U test and highlighted in bold to indicate a statistical significance or trend. NE: not evaluable.

Table S3. Predictive power of uEV transcripts to discriminate between stable disease and PCa risk reclassification.

**Transcript**	**AUC**	**95% CI**	***p* Value**
BCL2	0.561	0.426 to 0.696	0.378
BCL2L1 = BCL-XL	0.559	0.425 to 0.694	0.391
CCND1	0.541	0.405 to 0.677	0.551
DLX1	0.585	0.447 to 0.722	0.222
ERG	0.543	0.406 to 0.680	0.535
EZH2	0.556	0.421 to 0.692	0.416
HOXC6	0.613	0.483 to 0.743	0.103
KLK2	0.607	0.475 to 0.738	0.123
KLK3 = PSA	0.541	0.406 to 0.676	0.551
MCL1	0.518	0.384 to 0.652	0.798
NEAT1	0.609	0.477 to 0.740	0.115
PCAT1	0.555	0.422 to 0.689	0.423
PCAT14	0.552	0.414 to 0.690	0.453
PCGEM1	0.509	0.373 to 0.645	0.896
Prostein = SLC45A3	0.595	0.461 to 0.728	0.171
PSGR = OR51E2	0.554	0.418 to 0.690	0.436
PSMA = FOLH1	0.504	0.365 to 0.643	0.950
SChLAP1	0.558	0.418 to 0.698	0.403
SPDEF	0.544	0.408 to 0.681	0.521
STAT3	0.556	0.422 to 0.690	0.416
STAT5A	0.504	0.368 to 0.639	0.959
STAT5B	0.555	0.421 to 0.688	0.429
TMPRSS2	0.574	0.442 to 0.707	0.283
TRPM8	0.508	0.370 to 0.647	0.905

P values were calculated by ROC curve analysis. AUC: area under the curve, 95% CI: 95% confidence interval.
